# In Vitro Evaluation of Antipseudomonal Activity and Safety Profile of Peptidomimetic Furin Inhibitors

**DOI:** 10.3390/biomedicines12092075

**Published:** 2024-09-11

**Authors:** Sara Maluck, Rivka Bobrovsky, Miklós Poór, Roman W. Lange, Torsten Steinmetzer, Ákos Jerzsele, András Adorján, Dávid Bajusz, Anita Rácz, Erzsébet Pászti-Gere

**Affiliations:** 1 Department of Pharmacology and Toxicology, University of Veterinary Medicine, Hungary István utca 2, H-1078 Budapest, Hungaryjerzsele.akos@univet.hu (Á.J.); 2 Department of Laboratory Medicine, Medical School, University of Pécs, Ifjúság útja 13, H-7624 Pécs, Hungary; poor.miklos@pte.hu; 3 Molecular Medicine Research Group, János Szentágothai Research Centre, University of Pécs, Ifjúság útja 20, H-7624 Pécs, Hungary; 4 Department of Pharmacy, Institute of Pharmaceutical Chemistry, Philipps University, Marbacher Weg 6, 35032 Marburg, Germany; roman.lange@pharmazie.uni-marburg.de (R.W.L.);; 5 National Laboratory of Infectious Animal Diseases, Antimicrobial Resistance, Veterinary Public Health and Food Chain Safety, University of Veterinary Medicine, István utca 2, H-1078 Budapest, Hungary; 6 Department of Microbiology and Infectious Diseases, University of Veterinary Medicine, Hungária krt. 23-25, H-1143 Budapest, Hungary; 7 Medicinal Chemistry Research Group and Drug Innovation Centre, HUN-REN Research Centre for Natural Sciences, Magyar tudósok krt. 2, H-1117 Budapest, Hungary; 8 Plasma Chemistry Research Group, HUN-REN Research Centre for Natural Sciences, Magyar tudósok krt. 2, H-1117 Budapest, Hungary

**Keywords:** furin inhibitors, protein binding, oxidative stress, porcine intestinal cells, hepatocytes, cytochrome P450 3A4, *Pseudomonas aeruginosa*

## Abstract

Inhibitors of the serine protease furin have been widely studied as antimicrobial agents due to their ability to block the cleavage and activation of certain viral surface proteins and bacterial toxins. In this study, the antipseudomonal effects and safety profiles of the furin inhibitors MI-1851 and MI-2415 were assessed. Fluorescence quenching studies suggested no relevant binding of the compounds to human serum albumin and α_1_-acid glycoprotein. Both inhibitors demonstrated significant antipseudomonal activity in Madin–Darby canine kidney cells, especially compound MI-1851 at very low concentrations (0.5 µM). Using non-tumorigenic porcine IPEC-J2 cells, neither of the two furin inhibitors induced cytotoxicity (CCK-8 assay) or altered significantly the intracellular (Amplex Red assay) or extracellular (DCFH-DA assay) redox status even at a concentration of 100 µM. The same assays with MI-2415 conducted on primary human hepatocytes also resulted in no changes in cell viability and oxidative stress at up to 100 µM. Microsomal and hepatocyte-based CYP3A4 activity assays showed that both inhibitors exhibited a concentration-dependent inhibition of the isoenzyme at high concentrations. In conclusion, this study indicates a good safety profile of the furin inhibitors MI-1851 and MI-2415, suggesting their applicability as antimicrobials for further in vivo investigations, despite some inhibitory effects on CYP3A4.

## 1. Introduction

The world is currently experiencing a surge in the incidence of bacterial and viral diseases, many of which are likely to have zoonotic origins, such as severe acute respiratory syndrome coronavirus type 2 (SARS-CoV-2) and nosocomial infections by multidrug-resistant *Pseudomonas aeruginosa*. This has made the spread even more unpredictable, emphasising the need to develop new strategies to combat infectious microorganisms [1]. The progression of many infections depends on the host’s mechanisms, e.g., the activation of microbial proteins by host proteases such as transmembrane serine protease 2 (TMPRSS2), matriptase, or furin; hence, these enzymes could be relevant pharmacological targets to reduce or even completely stop the infectivity of pathogens.

Furin is a mammalian proprotein convertase and contains a subtilisin-like, calcium-dependent serine protease domain [2]. Due to the processing of numerous proproteins, it is involved in a multitude of physiological processes, beginning with embryonic development and extending to regulating many hormones and plasma proteins. Furthermore, it plays a role in the immune response, where it regulates T cell activity and is responsible for cytokine secretion, such as interferon-γ (IFN-γ), interleukin-4 (IL-4), and interleukin-13 (IL-13) [3]. It is therefore unsurprising that the damage or malfunction of furin contributes to the formation of pathological deviations in the mammalian organisms. It has been demonstrated that decreased furin expression or abnormally cleaved substrates may result in neurodegenerative disorders, such as Alzheimer’s disease [4], due to the accumulation of neurotoxic amyloid-β (Aβ) [5]. Furin-derived activation of growth factors and cytokines is also important in tumour progression, and can also lead to cardiovascular problems. Although not yet fully proven, there is strong evidence that altered furin activities play a role in the development and progression of metabolic diseases like type 2 diabetes mellitus [6].

Furin does not only exert effects on endogenous processes but it also has a significant impact on exogenous bacteria. It is known that furin is one of the most important proteases for the activation of numerous bacterial exotoxins, thus disabling the attachment of the toxin to the host cell. For example, the proteolytic activation of bacterial toxins has been demonstrated to promote the development of infections caused by *Pseudomonas aeruginosa* and *Bacillus anthracis* [7]. Multidrug-resistant *Pseudomonas aeruginosa*, which frequently causes nosocomial infections [8], harbors antibiotic resistance genes and exhibits a strong ability to form biofilms [9], making *Pseudomonas aeruginosa* resistant to environmental factors and conventional drug therapies in hospitals. This necessitated the exploration of new treatment methods to eliminate bacterial infections, including the use of phages and new antimicrobial compounds [10,11].

Additionally, certain viral strains require furin for viral entry into the host cell. Those viruses are primarily respiratory viruses, including the highly pathogenic avian influenza virus (HPAIV H5N1) or Middle East respiratory syndrome coronavirus (MERS-CoV). The cleavage of the S glycoprotein on the viral envelope of certain CoV to S1 and S2 subunits allows the virus to bind and fuse with the host cell, thereby promoting the infectivity and cell spread [3,12]. Inhibiting furin-like proprotein convertases could consequently lead to a decrease in viral pathogenicity, particularly when combined with the inhibition of other proteolytic enzymes such as TMPRSS2. Moreover, the inhibition of furin may also result in an increased immune response, as has been demonstrated in cell lines including human airway Calu-3 cells treated with the compound MI-1851 [13] or in a clustered regularly interspaced short palindromic repeats—associated protein 9 (CRISPR-Cas9) knockout cell line with adjusted genomic structure [14].

Currently, protein-based, peptide-based, peptidomimetic compounds, and small molecules with greater stability including guanidinylated aryl 2,5-dideoxystreptamine (GADDs) and BOS inhibitors are the primary means of inhibiting furin [15,16,17]. Oligo-arginine (Arg)-type inhibitors seem to be the appropriate lead compounds but they have been frequently modified in order to overcome their low stability [3,18]. The inhibitors within this group have already been assessed in vivo, with a focus on their antibacterial properties including antipseudomonal activity hexa-D-Arg amide in the ocular infection mouse model [7,19]. Another potent furin inhibitor is decanoyl-RVKR-chloromethyl ketone, a substrate analogue derivative that irreversibly binds to the catalytic site [3,18]. However, this compound type also exhibits significant limitations, including a lack of specificity and short half-life [20]. Furthermore, the covalent inhibitor QUB-F1, in which a diphenyl phosphonate warhead is connected to a substrate-like peptide RVKR, shows strong inhibitory potency against furin, but it lacks binding towards trypsin-like proteases [18]. A very potent furin inhibition has been also described for the peptidomimetic reversible derivatives MI-1851 (K_i_ for furin 10.1 pM, compound #8 [21]) and MI-2415 (K_i_ for furin 7.1 pM, compound #17 [22]), which only differ in their C-terminal P1-group. Thus, in the case of compound MI-1851, it is a 4-amidinobenzylamide (4-Amba) group, while in the case of inhibitor MI-2415, an aminoisoindole (Amia) has been incorporated.

In our study, the aim was to evaluate the safety profile of these two furin inhibitors, MI-1851 and MI-2415, in terms of their influence on cellular functions and protein binding capacity in addition to their potential protective effects against *Pseudomonas aeruginosa* infections using Madin–Darby canine kidney (MDCK) cells. Any alterations in cell viability and in the intracellular and extracellular redox status of the intestinal porcine enterocytes (IPEC-J2) and primary human hepatocytes (PHH), following 24 h treatment with the inhibitors, were also assessed. To exclude the possibility of drug interactions, the activity of the cytochrome P450 (CYP)3A4 isoenzyme was evaluated using a cell-based approach with PHH as well as a microsomal assay.

## 2. Materials and Methods

### 2.1. Spectroscopic Studies on Protein Binding of Furin Inhibitors

Human serum albumin (HSA) (product code: A1653) and α_1_-acid glycoprotein (AGP) (product code: G9885) were purchased from Merck (Darmstadt, Germany) and used as received. Fluorescence emission spectra were recorded using a Hitachi F-4500 fluorometer (Tokyo, Japan), while absorption spectra were collected by employing a Hitachi U-3900 UV–Vis spectrophotometer (Tokyo, Japan). Before the evaluation of fluorescence data, the inner-filter effects of MI-1851 and MI-2415 were corrected based on the following equation [23,24]:

(1) $$I_{cor}=I_{obs}\times e^{(A_{ex}+A_{em})/2}$$

where *I_cor_* and *I_obs_* are the corrected and observed emission intensities, respectively; and *A_ex_* and *A_em_* are the absorbance of the MI compounds tested at the excitation and emission wavelengths used, respectively.

To examine the potential interaction of inhibitor MI-2415 with HSA, increasing levels of the ligand (final concentrations: 0, 1, 2, 3, 4, 7, and 10 μM) were added to a standard amount of the protein (2 μM) in PBS (pH 7.4), and then the emission spectra were recorded (λ_ex_ = 295 nm, λ_em_ = 340 nm). Furthermore, the quenching effects of inhibitors MI-1851 and MI-2415 (both 0, 0.25, 0.5, 1, 1.5, 2, and 2.5 μM) were also tested on the emission signal of AGP (2 μM) in PBS (λ_ex_ = 285 nm, λ_em_ = 337 nm) [12,25]. Under the applied conditions, inhibitors MI-1851 and MI-2415 did not show any background fluorescence.

### 2.2. MDCK Cell Culturing and Pseudomonas aeruginosa Experiments

MDCK NBL-2 cells were obtained from Cytion (formerly: CLS Cell Lines Service, Eppenheim, Germany). Cells were grown in T-75 cell culture flasks, using Dulbecco’s Modified Eagle’s Medium (DMEM, Capricorn Scientific, Ebsdorfergrund, Germany) supplemented with 5% foetal bovine serum (FBS, Capricorn) and antibiotic–antimycotic solution (penicillin, streptomycin, amphotericin B; Merck, Darmstadt, Germany). The cells were subcultured and seeded in wells of 96-well microtiter plates one day prior to the experiments. After 24 h, near 100% confluence was recorded in the wells. The growth and incubation of MDCK cells were carried out in a humidified incubator at 37 °C and in the presence of 5% CO_2_. An overnight culture (18 h) of *Pseudomonas aeruginosa* was prepared on non-selective blood agar using a laboratory strain, isolated from a clinical case in 2022 (lab number: 730/22). Three times single colonies from ten different blood agar plates were dissolved in 5 mL of distilled water, and colony-forming units (CFU) were counted after their serial dilution and overnight incubation on non-selective blood agar at 37 °C. The repeated counting showed consistent bacterial counts in each colony after the same incubation time and conditions. The count of *Pseudomonas aeruginosa* from inoculating vials was also assessed and was in good accordance with our count prediction. The viability of MDCK cells was examined upon the addition of *Pseudomonas aeruginosa* (10^6^ CFU/mL) alone and in combination with inhibitor MI-1851, as well as compound MI-2415 at various concentrations (0.5, 1, 2.5, 5, 10, 25, 50, and 100 µM). These were added 2 h prior to and continuously during 5 h bacterial incubation. In accordance with the manufacturer’s instructions, a CCK-8 assay (Dojindo Molecular Technologies, Rockville, MD, USA) was performed. Following the addition of the final reagent (100 µL fresh Williams’ Medium E) and a 2 h incubation period, the absorbance was measured at 450 nm using a SpectraMax iD3 (Molecular Devices, San José, CA, USA).

### 2.3. IPEC-J2 and PHH Cell Culturing

The cell line used for the experiments was the IPEC-J2 from the jejunum of porcine. The IPEC-J2 epithelial cell line was a kind gift from Dr. Jody Gookin (Department of Clinical Sciences, College of Veterinary Medicine, North Carolina State University, Raleigh, NC, USA). The cell monolayers were maintained in 75 cm^2^ cell culture flasks with filtered caps (Orange Scientific, Braine-l’Alleud, Belgium) at 37 °C in a humidified atmosphere containing 5% CO_2_. The culture medium contained 50% Dulbecco’s modified Eagle’s medium (DMEM) and 50% Ham’s F12 Nutrient Mixture (Merck, Darmstadt, Germany) supplemented with 1.5 mmol/L HEPES, 5% fetal bovine serum (FBS) (Biocenter, Budapest, Hungary), 1% insulin/transferrin/sodium selenite medium supplement, 5 ng/mL epidermal growth factor (EGF), and 1% penicillin/streptomycin (all purchased from Invitrogen, Thermo Fisher Scientific, Waltham, MA, USA). Cells were used between passages 40 and 55. The media were changed every second day. Cells were seeded onto 96- and 24-well polystyrene cell culture plates for the cell viability and the Amplex Red/DCFH-DA assay, respectively.

PHHs were purchased from Primacyt Cell Culture Technology GmbH (Schwerin, Germany). Hepatocytes were thawed using hepatocyte recovery medium (Primacyt, Schwerin, Germany) and cells were centrifuged (10 min, 100× *g*, 20 °C) to remove supernatants. Cells were incubated with 5% FBS-containing plating medium for 6 h, then with FBS-free maintenance medium at 37 °C with 5% CO_2_ (both media were obtained from Primacyt, Schwerin, Germany) on Matrigel-coated membrane inserts (Costar Transwell-COL PTFE permeable supports, 6.5 mm inserts, 0.4 µm pore size, 24-well plate, Corning Incorporated, Kennebunk, ME, USA) for the CCK-8 and Amplex Red procedures. Maintenance medium without FBS was replaced every 24 h and the cells were incubated.

### 2.4. Cytotoxicity Measurements in IPEC-J2 and PHH-Based Cell Models

**Figure 1:**
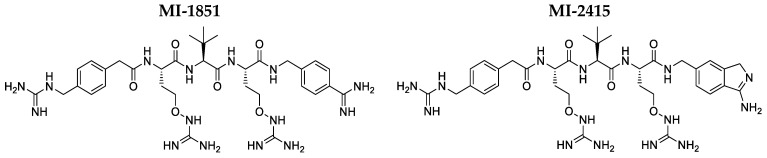
Chemical structures of the used furin inhibitors MI-1851 (**left** structure) [21] and MI-2415 (**right** structure) [22].

The possible cytotoxic effects of the inhibitors MI-1851 and MI-2415 (structures provided in Figure 1) in different concentrations on IPEC-J2 and those of inhibitor MI-2415 on PHHs were assessed for 24 h by performing the CCK-8 assay (Dojindo Molecular Technologies, Rockville, MD, USA). This assay uses the reducing capability of cellular dehydrogenase, measuring the appearance of nicotinamide adenine dinucleotide (NAD^+^) in its reduced form (NADH^+^H^+^), which acts on a water-soluble tetrazolium salt to form an orange-coloured formazan dye. IPEC-J2 cells were cultured on a 96-well plate and treated with compounds MI-1851 and MI-2415 at concentrations of 50 and 100 µM with three to four parallels, respectively. The plate was incubated at 37 °C for 24 h. Following three times washing of the wells with PBS, 10 µL CCK-8 reagent mixed with 100 µL fresh Williams’ Medium E was added and the absorbance values were measured at 450 nm using the SpectraMax iD3 microplate reader (Molecular Devices, San José, CA, USA) after 2 h incubation. The same method was applied with PHHs, exposed to inhibitor MI-2415 at 50 and 100 µM to the cells.

### 2.5. Amplex Red Measurements

To evaluate the cellular responses of IPEC-J2 to the inhibitors MI-1851 and MI-2415, and those of PHHs to MI-2415, at different concentration levels, the Amplex Red hydrogen peroxide assay kit (Invitrogen, Molecular Probes, Budapest, Hungary) in combination with horseradish peroxidase has been used. In the presence of hydrogen peroxide (in 1:1 stoichiometry), Amplex Red produces a red fluorescent oxidation product, resorufin. The IPEC-J2 cells and PHHs were treated for 24 h with the inhibitor(s) at different concentration levels (50 and 100 μM). After this treatment, 50 μL was collected from the cell supernatant and mixed with the same amount of Amplex Red working solution in each well. The cells were incubated at 37 °C for 30 min (with 5% CO_2_). The fluorometric assay was performed using a Victor X2 2030 (Perkin Elmer, Waltham, MA, USA) using an excitation wavelength of 530 nm and emission wavelength of 590 nm.

### 2.6. DCFH_2_-DA ROS Measurements

The ability of inhibitors MI-1851 and MI-2415 to induce oxidative stress (ROS) within IPEC-J2 as well as of compound MI-2415 within PHHs was investigated with 2,7-dichloro-dihydro-fluorescein diacetate (DCFH_2_-DA). DCFH_2_-DA is a non-fluorescent compound that can passively diffuse across cell membranes due to its lipophilic nature. Inside the cell, cellular esterase cleaves off the acetate group and, in the presence of H_2_O_2_, DCFH_2_ oxidizes to form a highly fluorescent compound, the 2,7-dichlorofluorescein (DCF). IPEC-J2 cells were challenged with inhibitors MI-1851 and MI-2415 and PHHs were treated with compound MI-2415 for 24 h at concentrations of 50 and 100 μM. Cells treated only with plain medium served as the control. The cells were washed twice with PBS three times and treated with 10 µM DCFH_2_-DA working solution for 30 min followed by rinsing with medium, scraping, and centrifugation for 10 min (at 3000× *g*). A Victor X2 2030 (Perkin Elmer, Waltham, MA, USA) was used to determine the fluorescence values of the samples (excitation wavelength: 485 nm, emission wavelength: 535 nm), which are directly proportional to the ROS generation.

### 2.7. CYP3A4 Activity Measurement and Modelling of Its Interaction with Furin Inhibitors

To investigate the effects of inhibitor MI-2415 on CYP3A4 activities, a P450-Glo CYP450 assay (Promega, Madison, WI, USA) was used. In this cell-based approach, PHHs were placed on 24-well plates coated with Matrigel and incubated with compound MI-2415 at concentrations of 25, 50, and 100 µM in three to six parallels. This luminescent method involves adding a cell-permeable Luciferin-IPA at 3 µM, which was used in 300 µL phenol-red free Williams’ Medium E in each well in the CYP3A4 assay with an incubation time of 1 h. The luminescence activity of the product in the presence of the luciferin detection reagent was proportional to the CYP activity. For the microsomal CYP3A4 assay of inhibitors MI-1851 and MI-2415 at 25, 50, and 100 µM (n = 3), BioVision CYP assays (BioVision, Inc., Kampenhout, Belgium) were used according to the manufacturer’s instructions. The protein content of human hepatic microsomes (Gibco, Biocenter, Szeged, Hungary) was determined by the bicinchoninic acid protein assay kit (Pierce BCA kit, Thermo Fisher Scientific, Waltham, MA, USA) and a 100 µg/well protein was used in each study. For the CYP3A4 assay, the selective positive control inhibitor ketoconazole (KCZ) was applied at 10 µM in each assay. A Victor X2 2030 fluorescence spectrometer (Perkin Elmer, Waltham, MA, USA) was used to detect the fluorescence intensities of the microsomal CYP3A4 function at wavelengths of λ_ex/em_ =  535/587 nm and it was also applied to detect the luminescence signals of the cell-based CYP3A4 activity assay.

For docking the small molecules into the active site of CYP3A4, the PDB structure 4K9W [26] was used. The ligands were prepared with Schrödinger Ligprep: briefly, protomers are generated in a pH range of 7.4 ± 1.5, and stereoisomers are enumerated for each ligand [27]. The ligands were then docked with Glide SP into the CYP3A4 structure [28,29]. The pKa values of the oxyguanidine groups of ligands MI-1851 and MI-2415 were calculated with Epik, according to the Hammett and Taft equations [30].

### 2.8. Statistical Analysis

The statistical analysis of the values was carried out using one-way ANOVA with 2023 R Core Team (R: A Language and Environment for Statistical Computing; R Foundation for Statistical Computing, Vienna, Austria) to determine differences between groups. Post hoc Tukey was applied for multiple comparisons. *p* < 0.05, *p* < 0.01, and *p* < 0.001 indicate statistically significant differences.

## 3. Results

### 3.1. Interactions of Inhibitors MI-1851 and MI-2415 with HSA and AGP

**Figure 2:**
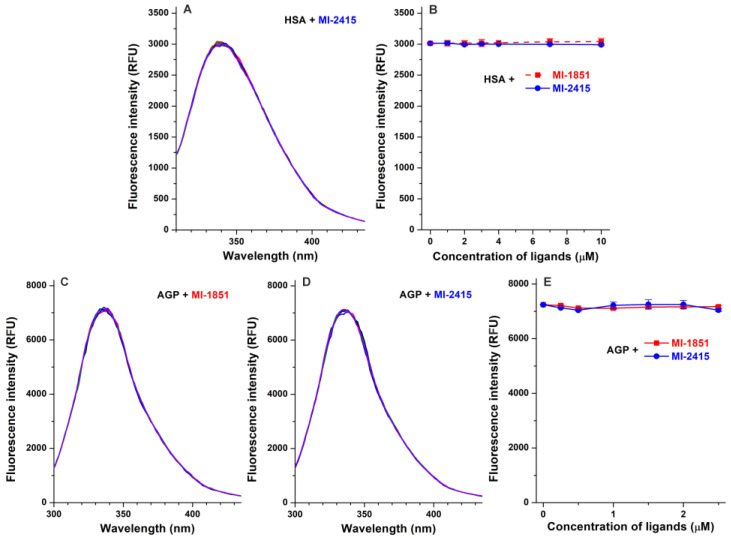
Effects of MI-1851 and MI-2415 on the fluorescence emission signals of HSA and AGP. Representative emission spectra ((**A**); λ_ex_ = 295 nm) and emission intensities ((**B**); λ_ex_ = 295 nm, λ_em_ = 340 nm; n = 3) of HSA (2 μM) in the absence and in the presence of increasing levels of inhibitor MI-2415 (1, 2, 3, 4, 7, and 10 μM) in PBS (pH 7.4). As it has been reported, also inhibitor MI-1851 did not affect the emission signal of HSA in the same experimental model (see in panel (**B**) with red dashed line) [12]. Representative emission spectra of AGP (2 μM) in the absence and in the presence of increasing concentrations (0.25, 0.5, 1, 1.5, 2, and 2.5 μM) of compounds MI-1851 (**C**) and MI-2415 (**D**) in PBS (pH 7.4; λ_ex_ = 285 nm). Influence of inhibitors MI-1851 and MI-2415 (means ± SEM) on the emission intensity of AGP ((**E**); λ_ex_ = 285 nm, λ_em_ = 337 nm; n = 3).

In our earlier study, compound MI-1851 did not affect the emission signal of HSA and the albumin binding of Site I and Site II marker drugs [12], suggesting no or only a very weak interaction with the protein. In the present work, the impact of analogue MI-2415 was also examined, where it did not influence the emission intensity of HSA at 340 nm (Figure 2A,B). Furthermore, both inhibitors MI-1851 and MI-2415 did not induce relevant changes in the fluorescence signal of AGP at 337 nm (Figure 2C–E).

### 3.2. Antipseudomonal Effect of Inhibititors MI-1851 and MI-2415

**Figure 3:**
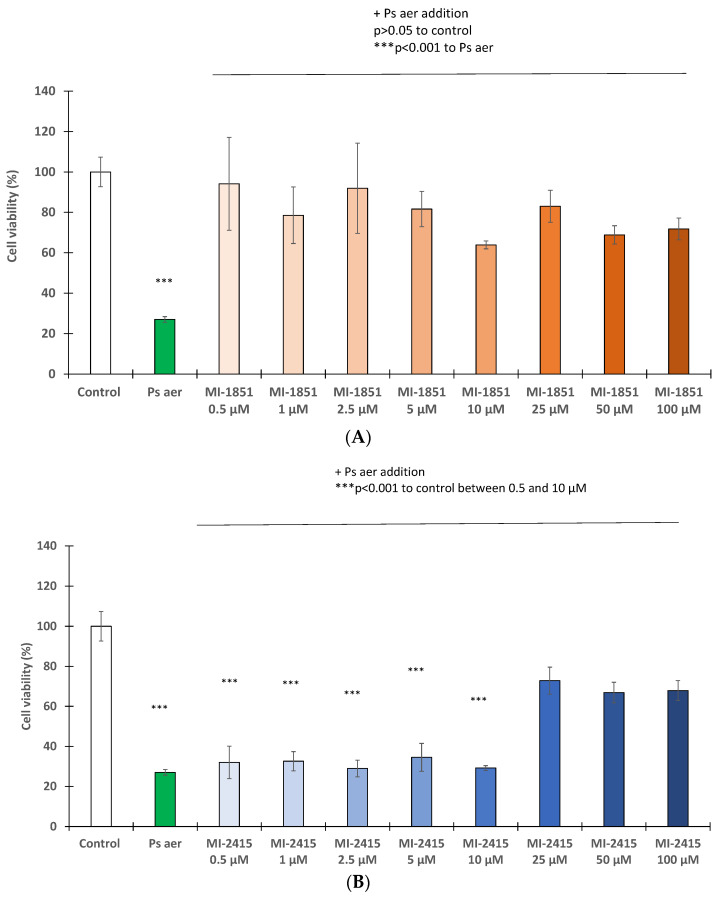
Determination of *Pseudomonas aeruginosa* (Ps aer) effect (10^6^ CFU/mL) on cell viability in MDCK cells with and without inhibitor MI-1851 (**A**) and MI-2415 (**B**) at different concentrations (0.5, 1, 2.5, 5, 10, 25, 50, and 100 µM). Furin inhibitors were added 2 h prior to and continuously during 5 h bacterial incubation. The data are the mean cell viability values expressed in percentage of control (%) ± standard errors of mean (SEM) (*** *p* < 0.001, *p* > 0.05; n = 4–8).

The viability of MDCK cells was significantly decreased by 5 h incubation with *Pseudomonas aeruginosa* at a concentration of 10^6^ CFU/mL (*p* < 0.001). However, treatment of the cells with inhibitor MI-1851 2 h prior to and simultaneously with bacterial challenge (Figure 3A) preserved cell viability, even at a concentration of only 0.5 µM (*p* > 0.05), as demonstrated in the CCK-8 assay. Similarly, the inhibitor MI-2415 (Figure 3B) also showed a prominent antipseudomonal effect at concentrations of 25 µM or above. It was also ascertained that significant differences were found between groups infected with *Pseudomonas aeruginosa* treated with MI-1851 (at 0.5 µM, *p* < 0.001) and MI-2415 (at 25 µM and above, *p* < 0.001) compared to those cells exposed only to the bacterial addition.

### 3.3. Cytotoxicity Investigations in IPEC-J2 Cells

**Figure 4:**
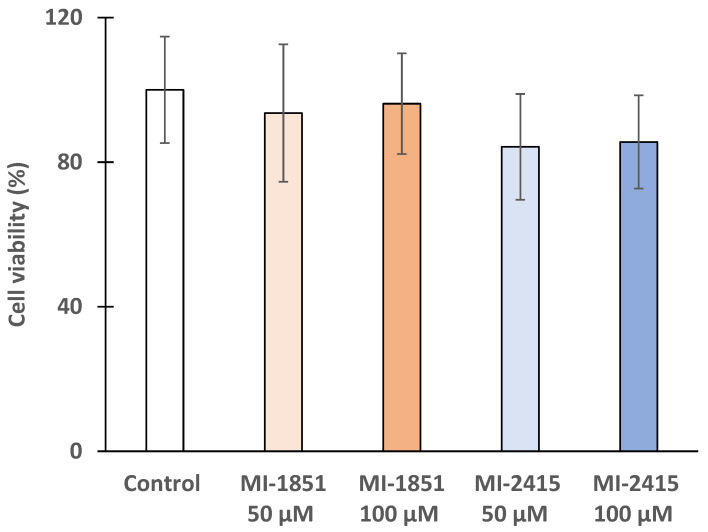
Cell viability after a 24 h treatment of IPEC-J2 cells with furin inhibitors MI-1851 or MI-2415 at 50 µM and 100 µM. The cell viability values are presented as percentage of control (%) ± standard errors of mean (SEM). Each group contained n = 3–4 samples (*p* > 0.05).

To measure the remaining quantity of viable cells in IPEC-J2 after the treatment with inhibitors MI-1851 and MI-2415 at 50 and 100 µM for 24 h, a CCK-8 assay was performed. The IPEC-J2 cell line did not show a significant decrease in cell viability (*p* > 0.05) upon the addition of both inhibitors at concentrations of 50 µM or 100 µM (Figure 4).

### 3.4. Peroxide Production in IPEC-J2 Cells

**Figure 5:**
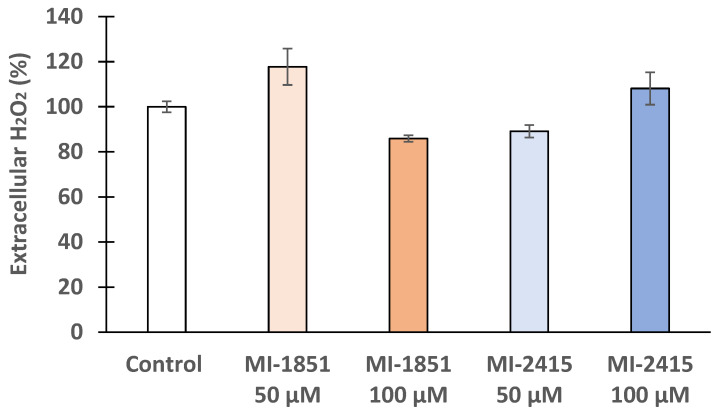
Effect of inhibitors MI-1851 and MI-2415 on extracellular H_2_O_2_ production. Both inhibitors were added at concentrations of 50 or 100 μM for 24 h on IPEC-J2 cells. Data represent the mean extracellular H_2_O_2_ (%) expressed in control ± standard errors of mean (SEM) by using the fluorescence readings (*p* > 0.05; n = 4).

The extracellular redox status of IPEC-J2 cells after their treatment with furin inhibitors MI-1851 and MI-2415 was assessed, using an Amplex Red hydrogen peroxide assay kit. Our findings illustrate that the applied inhibitors did alter their cell redox balance slightly, but statistically not significantly compared to the control, even at very high concentrations of 100 μM (*p* > 0.05) (Figure 5).

### 3.5. DCFH-DA Assay to Detect Peroxide Production in IPEC-J2 Cells

**Figure 6:**
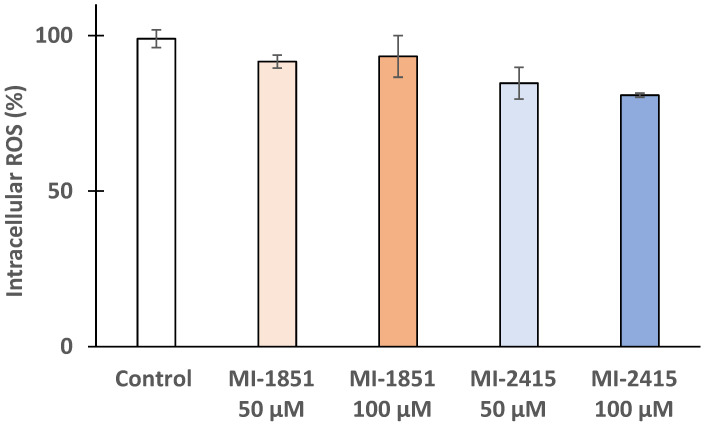
Effects of inhibitors MI-1851 and MI-2415 at different concentrations (50 and 100 μM) on intracellular oxidative stress on IPEC-J2 cells. The data represent the mean intracellular ROS (%) expressed in control ± standard errors of mean (SEM) (*p* > 0.05; n = 4).

To monitor any significant changes in oxidative stress levels within the IPEC-J2 cells, the DCFH-DA assay was performed. Figure 6 shows no significant alterations in intracellular ROS levels (*p* > 0.05) after the administration of inhibitors MI-1851 and MI-2415.

### 3.6. Viability and Redox Status of Hepatocytes Exposed to Inhibitor MI-2415

**Figure 7:**
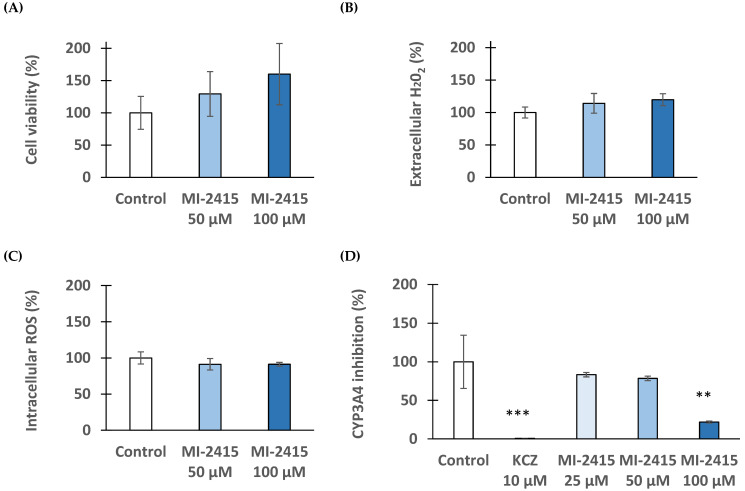
Effects of inhibitor MI-2415 at different concentrations (50 and 100 μM) on cell viability ((**A**), CCK-8), extracellular ((**B**), Amplex Red) and intracellular ((**C**), DCFH-DA) oxidative stress, and on CYP3A4 activity (**D**) in PHHs compared to control. The data represent the mean measured values (%) expressed in percentage of control ± standard errors of mean (SEM) (*** *p* < 0.001, ** *p* < 0.01, *p* > 0.05; n = 3–6). Ketoconazole (KCZ) at 10 µM was used as reference CYP3A4 inhibitor.

CKK-8 assay was performed with PHHs upon the addition of inhibitor MI-2415 at concentrations of 50 µM and 100 µM for 24 h (Figure 7A). The inhibitor did not lead to significant cell death even at high concentrations of 100 µM (*p* > 0.05). It was also ascertained that the inhibitor did not cause a redox imbalance in PHHs exposed to 50 µM and 100 µM based on measurements of extracellular H_2_O_2_ production and intracellular ROS formation (*p* > 0.05) (Figure 7B,C). CYP3A4 activity was significantly reduced in PHHs exposed to inhibitor MI-2415 at 100 µM for 24 h (*p* = 0.00313, Figure 7D). Ketoconazole (KCZ), used as a reference compound (at 10 µM), could greatly suppress the CYP3A4 function (*p* < 0.001).

### 3.7. Microsomal CYP3A4 Activity Assay

**Figure 8:**
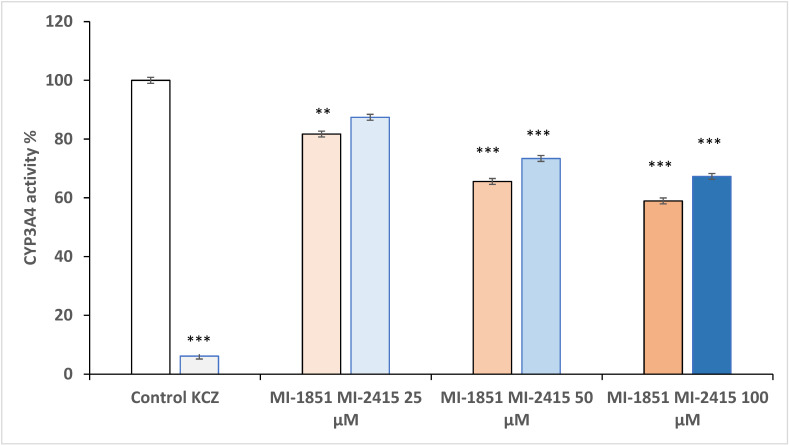
Effects of inhibitors MI-1851 and MI-2415 at concentrations of 25, 50, and 100 µM on microsomal CYP3A4 function. The data represent the mean CYP3A4 activity (%) expressed in percentage of control ± standard errors of mean (SEM) (*** *p* < 0.001, ** *p* < 0.01, *p* > 0.05; n = 3). Ketoconazole (KCZ) at 10 µM was used as reference CYP3A4 inhibitor.

Human microsomal data previously showed that compound MI-1851 (in an elevated concentration range of 25–100 µM) suppressed CYP3A4 function in a concentration-dependent manner (*p* = 0.071 at 25 µM and *p* < 0.001 at 50 µM and 100 µM). The other furin inhibitor, MI-2415, could also reduce CYP3A4 activity but only from 50 µM (in both cases *p* < 0.001). The reference inhibitor, KCZ, at 10 µM could decrease the CYP3A4 isoenzyme to the highest degree (*p* < 0.001) (Figure 8).

### 3.8. Molecular Modelling of MI-1851 and MI-2415 with CYP3A4

**Figure 9:**
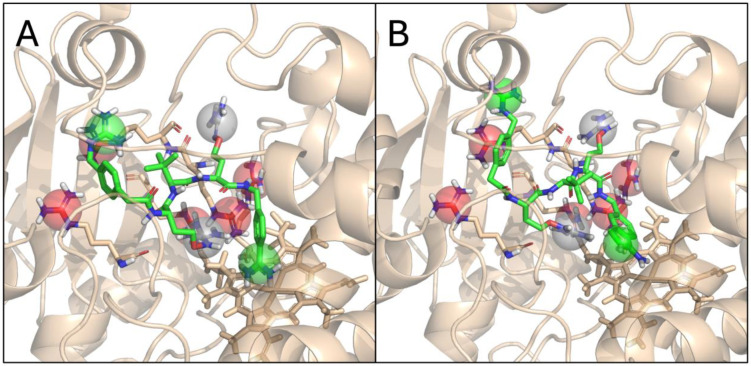
Predicted binding modes for MI-1851 (**A**) and MI-2415 (**B**) in the active site of CYP3A4. While the site is big enough to fit the ligands, the cluster of positively charged arginine residues (red spheres) produces a repulsive force against the positively charged groups of the ligands (green spheres). The canavanine groups (grey spheres) have a pKa value close to 7, meaning that they can be present in the protonated and deprotonated forms as well. This explains the intermediate level of CYP3A4 inhibition, as the most heavily charged (+4) protomers are unlikely to bind, while the mildly charged forms (+2) are easily accommodated.

In our previous work, we investigated a series of peptidomimetic host matriptase/TMPRSS2 inhibitors for their safety profiles [31]. Notably, the whole series had a measurable inhibitory activity against CYP3A4 (but not other CYP isoforms) with the exception of one congener, MI-21. We have rationalized this observation based on ligand docking into the two available binding pockets (active site and peripheral pocket), as well as the number of positively charged groups in the members of this series, concluding that the observed inhibitory activities can be explained by assuming the active site as the primary location of binding. In particular, we proposed that the more heavily charged ligand MI-21 (with three positively charged groups) experiences a larger repulsive force from a tight cluster of five arginine residues at the active site than the rest of the series with one or two positive charges. The intermediate level of CYP3A4 inhibition detected for MI-2415 and MI-1851 at 100 µM is in line with this observation, if we account for the unique nature of the oxyguanidine groups of the two canavanine side chains of each ligand. Specifically, the pKa value of this moiety is very close to 7 (the literature: 7.01 [32], own calculations: 7.48–7.77), meaning that both the acidic (positively charged) and basic (neutral) protomers are present in the solution. This in turn means that the number of positively charged groups ranges between 2 and 4 under physiological conditions, which explains the CYP3A4 inhibitory activities between 30 and 50% (Figure 9).

## 4. Discussion

IPEC-J2 cells, derived from piglet jejunum, are non-transformed and exhibit characteristics of mature enterocytes, forming a polarized monolayer crucial for studying normal intestinal physiology and pathogen interactions [33], due to the presence of tight junctions, microvilli, and a glycocalyx [34]. This non-tumorigenic nature contrasts with tumorigenic cell lines such as Caco-2 and HT-29, which, although also forming polarized monolayers, display cancer-associated traits that influence their utility in research aiding in drug permeability and infection mechanism studies [33,35]. However, their cancerous properties can limit their effectiveness in modelling normal intestinal physiology [33,36]. Similarly, a human liver cancer HepG2 cell line significantly differs from PHHs, which retain more in vivo-like metabolic functions and responses [36]. IPEC-J2 and PHHs, both used in this study, offer physiologically relevant models for studying normal functions and responses.

In previous studies, the incorporation of *tert*-leucine (Tle) P3 residues in Amba-derived furin inhibitors with an additional basic P5 group led to the development of the inhibitor 4-guanidinomethyl-phenylacetyl-Arg-Tle-Arg-4-Amba (MI-1148, K_i_ = 5.5 pM), which showed excellent antiviral properties against canine distemper virus and highly pathogenic avian influenza viruses (HPAIVs), H5N1 and H7N1 [37], as well as against West-Nile and dengue-2 virus [38], respiratory syncytial virus (RSV) [39], and mumps virus [40]. An antibacterial effect via the inhibition of anthrax and diphtheria toxin-related action in various cell culture assays could also be observed [37]. Although MI-1148 also possessed high stability in human plasma, it showed limited safety in toxicity studies in mice; the maximum tolerated dose was found to be 2.5 mg/kg after intraperitoneally (i.p.) treatment. At the next higher dose of 5 mg/kg i.p., all four mice died. Furthermore, when testing the toxicity of several compounds, we could not find a close correlation between the inhibitory strength against furin and the toxicity. Therefore, we speculated that the toxicity is not caused by the inhibition of furin itself, but by addressing a so far unknown off-target. Moreover, the strongest toxicity was observed for the most basic compound, e.g., inhibitor MI-1148, which contains four strongly basic groups, the guanidines on the P2, P4, and P5 residues, respectively, as well as the amidine on the P1 group [41].

In later studies, we found that the replacement by the strongly basic Arg residues in the P2 and P4 position by the structurally closely related less basic canavanine (pKa of its oxyguanidine side chain reduced to 7.01) provided inhibitors with significantly reduced toxicity, while still maintaining a very strong in vitro furin inhibition in enzyme kinetic studies and also high antiviral potency in cell culture. In the case of inhibitor MI-1851, the mice tolerated an i.p. dose of 15 mg/kg (no higher dose was tested) [21,42]. Furthermore, in the case of inhibitor MI-2415, the P1 Amba residue used in inhibitor MI-1851 was replaced by the Amia residue, while keeping the canavanines in the P2 and P4 position. Although the basicity of the Amia group is slightly reduced compared to the amidine in the Amba residue, it had no further beneficial effect on the toxicity profile of the compound. The mice also tolerated an i.p. dose of 15 mg/kg of inhibitor MI-2415, whereas one of four mice died at the next higher dose of 20 mg/kg [22].

We also speculated that the decreased toxicity found for the canavanine containing inhibitors MI-1851 and MI-2415 is caused by a reduced partial protonation of the inhibitor’s P2 and P4 side chains in the bloodstream at pH 7.4, leading to a reduced affinity to the unknown off-target. In contrast, for antiviral activity, the furin located in the trans-Golgi network (TGN) must be addressed, where the canavanine side chains of the inhibitors should be widely protonated due to the reduced pH close to 6 [21]. Very recently, we proposed the Mas-related G protein–coupled receptor X2 (MRGPRX2) as the potential off-target for these peptidomimetic furin inhibitors and could demonstrate a much stronger activation of this mast cell receptor by the more toxic inhibitor MI-1148 compared to the less toxic analogues MI-1851 and MI-2415 [22]. However, further experiments are required to confirm this hypothesis.

Several developed substrate analogues, 4-Amba-type furin inhibitors with phenylacetyl-Arg-Val-Arg moiety, were also prepared and tested potent against furin-dependent pathogens such as diphtheria toxin intoxication in African green monkey kidney epithelial Vero cells and a hamster kidney cell infected with Semliki Forest virus [43].

To exclude any direct effects of the furin inhibitors MI-1851 and MI-2415 on cell viability, a cell-based CCK-8 assay was conducted to evaluate their potential cytotoxicity. So far, no studies have evaluated the impact of MI-2415 in any intestinal and liver cell line model yet. For the investigations, IPEC-J2 as well as PHHs were selected mainly due to their ability to resemble in vivo-like conditions, as previously described [36]. Even at a concentration of 100 µM and an incubation time of 24 h, no significant cell death was observed after treatment with MI-1851 and MI-2415. The non-cytotoxic effect of MI-1851 at up to 100 µM has already been described in PHHs [42] and up to 50 µM in human airway Calu-3 cells [13].

During infection with pathogens, cells are often exposed to elevated concentrations of reactive oxygen species (ROS), leading to the oxidation of proteins, lipids, and nucleic acids and inhibition of physiological cell function [44,45,46]. Consequently, it is important that potential therapeutic strategies should not induce further oxidative stress in host cells. The effects of both inhibitors were examined on the intracellular and extracellular hydrogen peroxide production and it was ascertained that neither of the two inhibitors induced a significant increase in ROS levels at a concentration of 100 µM in the two cell lines employed. It was previously established that inhibitor MI-1851 could be safely used in PHHs at the same concentration, lacking any significant effect on extracellular hydrogen peroxide production [42]. Taken together, no redox imbalance can be expected when the furin inhibitors MI-1851 or MI-2415 are used on their own in the liver–gut axis.

The formation of stable ligand–HSA or ligand–AGP complexes may have a considerable effect on the pharmacokinetics of drugs [47,48]. Fluorescence quenching experiments are frequently applied to examine ligand–protein interactions: Since the fluorescence signal of tryptophan residues is sensitive to microenvironmental changes, the complex formation of a ligand with a protein typically influences the emission signal of the macromolecule [49,50,51]. Previous quenching experiments demonstrated that certain protease inhibitors (e.g., inhibitors of numerous trypsin-like serine proteases such as MI-472 and MI-477) form weak to moderate interactions with HSA and relatively stable complexes with AGP, while other derivatives (such as compound MI-21) did not bind to these plasma proteins [12,25]. So far, there is no information about the interaction of 1*H*-isoindol-3-amine type furin inhibitors and HSA/AGP binding available. Nevertheless, our earlier [42] and current results suggest that inhibitors MI-1851 and MI-2415 do not form stable complexes with HSA or AGP.

In the present study, we investigated the effects of both MI-1851 and MI-2415 against *Pseudomonas aeruginosa* using MDCK cell lines. Previous studies revealed promising antipseudomonal effects in preclinical studies with certain furin inhibitors, including nona-D-Arg amide (D9R) [52] and the peptidomimetic compound peptidyl decanoyl-RVKR-chloromethyl-ketone [3] small molecules such as GADDs and BOS inhibitors, known for a lack of requirement for proteolytic decomposition [17,53]. Our work unequivocally proves that both Amba- and Amia-type furin inhibitors exhibit potent antipseudomonal activity without affecting the viability of cells up to a concentration of 100 µM. Our results indicate that inhibitor MI-1851 can be safely applied at any of the tested concentration levels down to 0.5 µM, and MI-2415 showed a safe profile at concentrations of 25 µM or higher. In light of these findings, especially inhibitor MI-1851, being much more active than MI-2415, could be used to address the global rise of new forms of drug-resistant *Pseudomonas aeruginosa*.

For the evaluation of the potential drug interactions, the impact of inhibitors MI-1851 and MI-2415 on CYP3A4 was investigated, which is one of the major human liver cytochromes. Using human hepatic microsomes, a concentration-dependent inhibition of CYP3A4 for both inhibitors was observed, starting to be significant with a concentration of 25 µM for MI-1851 and 50 µM in the case of MI-2415. In PHHs, a significant suppression of CYP3A4 activity was only detectable at 100 µM upon the addition of inhibitor MI-2415. In previous studies with PHH treated with inhibitor MI-1851, a significant inhibition of CYP3A4 was already observed at a concentration of only 20 µM [42]. The minor structural difference of the P1 group (Amba in the case of compound 1851 and Amia in inhibitor MI-2415) must be responsible for their extent on CYP3A4 modulation at different concentrations. The requirement for higher inhibitor doses to see the effects of inhibitors MI-1851 and MI-2415 on CYP3A4 function in hepatocytes-based models compared to those in microsomes might be caused by the poor cell membrane permeability of these tetrabasic furin inhibitors. In contrast, less polar monobasic or dibasic amidinophenylalanine-derived inhibitors, such as MI-432, MI-463, MI-482, and MI-1900, have been demonstrated to inhibit CYP3A4 already at considerably lower concentrations [12]. Thus, it can be concluded that MI-2415 especially, but also MI-1851, only induces meaningful changes at exceedingly high doses. We have explained this difference with our earlier hypothesis on the mentioned series, namely that the repulsive force between the positively charged groups of the inhibitors and a cluster of five arginine residues at the active site of CYP3A4 hinders binding and, consequently, CYP3A4 inhibition for peptidomimetics with a larger number of positively charged sidechains. For the specific case of MI-1851 and MI-2415, an intermediate level of CYP3A4 inhibition is observed, consistently with the variable protonation state of the two canavanine sidechains, resulting in an overall charge ranging from +2 to +4 for these peptidomimetics. Consequently, a relatively low risk of drug interactions can be expected when the furin inhibitors investigated in this study would be administered at therapeutic doses. Especially, for inhibitor MI-1851, a low dose of approximately 1 µM should be sufficient to obtain a significant protective effect again *Pseudomanas aeruginosa* intoxications.

A new era has emerged with the development of this slight pH difference-regulated protonation-driven compartment-specific action of furin inhibitors. The diverse chemical properties at P1 influence the polarity and the cell permeability, with novelly synthesized Amia-type MI-2415 exhibiting a more hydrophobic character, which enhances its capacity to cross cell membranes [22] compared to its Amba analogue, MI-1851. In our study, it was firstly reported that these types of furin inhibitors have beneficial effects against *Pseudomonas aeruginosa*-caused infection in the MDCK cell-based model, pointing out the repurposing potential of antiviral furin blockers as antibacterial agents.

In conclusion, for discovering new antimicrobial compounds, in vitro safety evaluation is crucial to identify possible side effects prior to in vivo applications. For the furin inhibitors MI-1851 and MI-2415, no significant impact on cell death, oxidative stress, or protein binding was observed in either IPEC-J2 or PHHs. The concentration-dependent inhibition of CYP3A4 activity was present only at higher concentrations. In our work, Amba- and Amia-derived furin inhibitors showed both a significant antipseudomonal effect, despite MI-1851 being effective at much lower concentrations. Moreover, other bacterial toxins such as the anthrax toxin, aerolysin toxin, shiga toxin, and diphtheria toxin also require activation by furin, besides *Pseudomonas aeruginosa* exotoxin A (PEA) intoxication. The complete antimicrobial spectra of these furin inhibitors have to be investigated in future studies.

## Data Availability

All raw data supporting the results of the present study can be achieved from the corresponding author upon reasonable request.
